# Mechanisms underlying capsulotomy for refractory obsessive-compulsive disorder: neural correlates of negative affect processing overlap with deep brain stimulation targets

**DOI:** 10.1038/s41380-023-01989-1

**Published:** 2023-03-06

**Authors:** Hailun Cui, Yingying Zhang, Yijie Zhao, Ying Zhao, Qiong Ding, Ruiqin Chen, Luis Manssuer, Chencheng Zhang, Wenjuan Liu, Dianyou Li, Bomin Sun, Valerie Voon

**Affiliations:** 1 Department of Psychiatry, University of Cambridge, Cambridge, United Kingdom; 2 Department of Neurosurgery, Center for Functional Neurosurgery, Ruijin Hospital, Shanghai Jiao Tong University School of Medicine, Shanghai, China; 3 Neural and Intelligence Engineering Centre, Institute of Science and Technology for Brain-Inspired Intelligence, Fudan University, Shanghai, China; 4 Department of Psychological Medicine, Zhongshan Hospital, Fudan University, Shanghai, China; 5 Cambridgeshire and Peterborough NHS Foundation Trust, Cambridge, United Kingdom

**Keywords:** Neuroscience, Psychiatric disorders

## Abstract

Ablative procedures such as anterior capsulotomy are potentially effective in refractory obsessive-compulsive disorder (OCD). Converging evidence suggests the ventral internal capsule white matter tracts traversing the rostral cingulate and ventrolateral prefrontal cortex and thalamus is the optimal target for clinical efficacy across multiple deep brain stimulation targets for OCD. Here we ask which prefrontal regions and underlying cognitive processes might be implicated in the effects of capsulotomy by using both task fMRI and neuropsychological tests assessing OCD-relevant cognitive mechanisms known to map across prefrontal regions connected to the tracts targeted in capsulotomy. We tested OCD patients at least 6 months post-capsulotomy (*n* = 27), OCD controls (*n* = 33) and healthy controls (*n* = 34). We used a modified aversive monetary incentive delay paradigm with negative imagery and a within session extinction trial. Post-capsulotomy OCD subjects showed improved OCD symptoms, disability and quality of life with no differences in mood or anxiety or cognitive task performance on executive, inhibition, memory and learning tasks. Task fMRI revealed post-capsulotomy decreases in the nucleus accumbens during negative anticipation, and in the left rostral cingulate and left inferior frontal cortex during negative feedback. Post-capsulotomy patients showed attenuated accumbens-rostral cingulate functional connectivity. Rostral cingulate activity mediated capsulotomy improvement on obsessions. These regions overlap with optimal white matter tracts observed across multiple stimulation targets for OCD and might provide insights into further optimizing neuromodulation approaches. Our findings also suggest that aversive processing theoretical mechanisms may link ablative, stimulation and psychological interventions.

## Introduction

Obsessive-compulsive disorder (OCD) is a common psychiatric condition and a leading cause of disability with a significant proportion remaining treatment refractory. Surgical interventions have shown potential efficacy in selected patients with refractory OCD. Both deep brain stimulation (DBS) targeting the anterior limb of the internal capsule (ALIC), subthalamic nucleus (STN) or bed nucleus of the stria terminalis (BNST) and ablative surgeries targeting the cingulum bundle or ALIC have shown efficacy [1–6]. A recent study suggested potentially greater efficacy in ablative surgery with 50.4% improvement in obsessive-compulsive symptoms relative to 40.9% in DBS, along with a lower rate of adverse events [7]. The therapeutic mechanism underlying anterior capsulotomy is based on the circuit-based hypotheses of OCD pathophysiology disrupting the frontostriatal white matter tracts traversing ALIC and thus interrupting the bidirectional communication between key structures in the prefrontal cortex (i.e., anterior cingulate cortex (ACC), orbitofrontal cortex (OFC), ventral medial prefrontal cortex (vmPFC), and lateral prefrontal cortices) and subcortical areas (striatum and thalamus) [8, 9]. Converging evidence suggests the ventral internal capsule white matter tracts traversing the rostral cingulate and ventrolateral prefrontal cortex and thalamus is the optimal target for clinical efficacy across multiple DBS targets for OCD [10]. The white matter tracts targeted in capsulotomy overlaps with ALIC DBS targeting [11]. The question of which white matter tracts and potential cognitive processes associated with the prefrontal connections to target is relevant to precision ablative targeting to optimize outcomes, particularly with the advent of novel techniques such as MR-guided focused ultrasound (MRgFUS) [12].

Several compelling theoretical cognitive mechanisms have been proposed for OCD which is characterized by obsessions or intrusive repetitive thoughts provoking anxiety and compulsions or rituals performed to alleviate the anxiety. A leading theory hypothesized to underlie the highly effective psychological intervention of exposure response therapy for OCD is that of aversive conditioning and extinction [13–16]. This theory posits that the obsessions and compulsions may be related to the anticipation and processing of aversive threat [17, 18], and particularly the avoidance of aversive threat [19]. In healthy individuals, emotional reactivity facilitates adaptive function that promotes the efficient allocation of brain resources to approach or avoid future events [20]. In OCD patients, the anticipation of a potential aversive outcome might enhance anxiety-generating obsessions through aberrant fear and anxiety conditioning with hypervigilance for aversive stimuli and aberrant avoidance of aversive stimuli [21–23]. A related phenomenon is the potential impairment in the learning of safety signals which indicates that a future threat is non-existent and may take the form of an appetitive or neutral context [24].

Cognitive processes have well-established anatomy that are translationally relevant from animal to human studies and represent the cornerstone of the National Institute of Mental Health Research Domain Criteria (NIMH RDoC) as potential objective biomarkers for therapeutic prediction and outcomes [25]. Here, we sought to ask if capsulotomy might be associated with functional remodelling of neural substrates related to aversive processing by comparing OCD patients who have undergone capsulotomy at least six months previously with matched OCD controls and healthy controls. We used a modified version of the monetary incentive delay (MID) task investigating the anticipation and experience of uncertain negative affective stimuli along with a within session extinction trial. Meta-analyses of the MID task implicate the ventromedial prefrontal cortex (vmPFC) and nucleus accumbens (NAc) as key regions [26–28]. Furthermore, aversive conditioning and extinction implicate the dorsal cingulate and vmPFC, respectively [29–31]. This study was part of a larger study in which we investigated which prefrontal regions and underlying cognitive processes might be implicated in the effects of capsulotomy by using both functional MRI tasks and cognitive tasks assessing OCD-relevant cognitive mechanisms known to map across prefrontal regions connected to the ALIC tracts targeted in capsulotomy. In addition to the negative affect task reported here, the additional fMRI tasks included tasks assessing set shifting (ventrolateral prefrontal cortex), reversal (orbitofrontal cortex), uncertainty (dorsal cingulate), working memory (dorsolateral prefrontal cortex) and conflict (dorsal cingulate). As there was no obvious difference as a function of capsulotomy and OCD controls in these other tasks, we focus on the negative affect task reported here with the results of other fMRI tasks reported elsewhere. The neuropsychological tests reported in this study examined working memory, planning, set shifting, inhibition, associative learning and attention which have been shown to be impaired in OCD [32, 33]. These constructs have well-established prefrontal-subcortical substrates with the tasks selected specifically intended to parse the differing prefrontal brain regions implicated in OCD to ask which might be influenced by capsulotomy.

## Methods and material

### Participants

OCD patients who underwent capsulotomy surgery (CAP), age- and gender-matched OCD controls (OCDc), and healthy controls (HC) were recruited from the outpatient clinic/institutional database at Ruijin Hospital, Shanghai Jiao Tong University School of Medicine. OCD diagnosis was established using the Structured Clinical Interview for DSM-V by senior psychiatrists. OCD symptoms were assessed with the clinician-rated Yale–Brown Obsessive–Compulsive Scale (Y-BOCS). Inclusion/exclusion criteria and the capsulotomy procedure are described in the Supplement and previous publications [34]. The CAP patients were scanned at least six months post-surgery to allow symptom improvement to stabilize (see Fig. S1 for lesion locations of each patient). Subjects were right-handed, had normal or corrected-to-normal vision and provided written informed consent prior to their participation in the research. All study procedures were approved by the Institutional Review Board of Ruijin Hospital, Shanghai Jiao Tong University School of Medicine (No. 2019-197).

### Clinical and cognitive assessments and statistical analyses

Participants also completed the patient-rated Beck Depression Inventory (BDI), the Quality of Life Enjoyment and Satisfaction Questionnaire-Short Form (Q-LES-Q-SF), the Sheehan Disability Scale (SDS), the Hamilton Depression Rating Scale (HAMD) (17-item version) and the Hamilton Anxiety Scale (HAMA).

Selected tests from the Cambridge Neuropsychological Automated Test Battery (CANTAB) known to be impaired in OCD [32, 33] were tested including the Intra-Extra Dimensional Set Shift task (IED), the Stop Signal Task (SST), the Stockings of Cambridge task (SOC), the Spatial Working Memory task (SWM), the Paired Associative Learning task (PAL), the Rapid Visual Information Processing task (RVP), and the Pattern Recognition Memory task (PRM) to measure cognitive flexibility, response inhibition, goal-directed planning, visuospatial working memory, visual memory, sustained attention and visual pattern recognition memory, respectively.

For demographic information and clinical measures, we applied χ2 test or one-way ANOVA to assess group differences. For a subset of the CAP group with pre-capsulotomy clinical assessments in Y-BOCS, HAMA and HAMD, we applied a paired t-test to examine capsulotomy-induced effects before and after the surgery. Cognitive outcome measures were assessed for normality of distribution and outliers and skewed data in percent format were first arcsine transformed. Cognitive data were analysed using one-way ANOVA, or the Kruskal-Wallis test to detect group differences (significance assigned after Bonferroni correction for multiple comparisons (*p* < 0.005), followed by Tukey’s test or Dunn’s test for post hoc analyses). Cognitive measures showing significant group differences unadjusted for covariates were further examined using one-way analysis of covariance (ANCOVA) with group as fixed factor and age, gender, years of education, and BDI as covariates. Y-BOCS was also added as a covariate when a post-hoc group difference was found between OCDc and CAP. We removed 3 SST outliers in the OCDc group (defined as 1·5 × IQR rule from the mean of each group). Cohen’s d as a measure of effect size was calculated for significant *t*-test results, and partial eta squared (η_p_^2^) for ANOVA models. All statistical analyses were conducted using R Statistical Software (Version 4.0.3; R Foundation for Statistical Computing, Vienna, Austria).

### fMRI task: aversive avoidance task (AV) and aversive extinction task (EV)

**Fig. 1:**
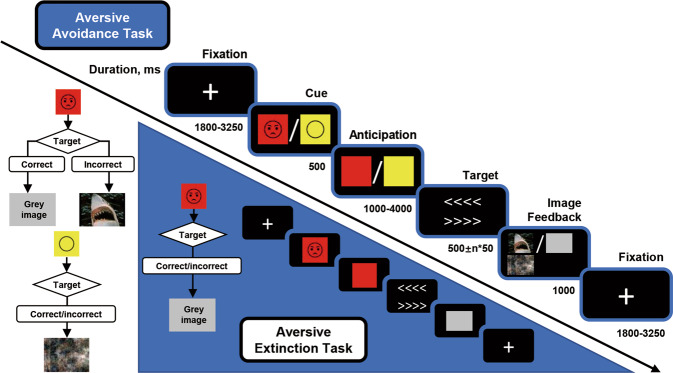
Task design: aversive avoidance task (AV) and aversive extinction task (EV). In the AV task, two different cues (either aversive or neutral) indicating the valence of anticipation (AV_aversive/AV_neutral) were followed by three types of feedback images (AV_aversive/AV_grey/AV_neutral), with a target slide indicating the direction of button press in between. During aversive conditions, participants were shown grey images for correct and rapid response to the target slides, and negative images for incorrect or no response. During neutral conditions, neutral images were shown regardless of correctness or rapidity of responses. In the EV task, cues with single valence indicating aversive anticipation (EV_aversive) were followed by grey feedback images (EV_grey) regardless of correctness and rapidity of responses against target slides. The AV paradigm consists of 60 trials (with 30 aversive or neutral trials each presented in random order) in total, while the EV consists of 50. Note: Example for negative and neutral pictures is taken from the International Affective Picture System (IAPS).

The first fMRI aversive avoidance task (AV) (Fig. 1) was adapted from the monetary incentive delay task (MID) [35], which dissociates the anticipation and feedback phase. Briefly, one of two visual cues indicated that the outcome feedback image would be either aversive or neutral. The 500 ms cue was followed by a 1000–4000 ms jittered delayed anticipatory period and a target arrow indicating the direction of the button press. The outcome feedback image displayed for 1000 ms is dependent on the correctness and rapidity of responses. In the aversive avoidance condition, participants were shown grey images for correct and timely response, and negative images for incorrect or no response. In the neutral condition, neutral images were shown regardless of correctness and timely response. The images were obtained from International Affective Picture Set (IAPS) [36]. This task consisted of 30 aversive and 30 neutral trials presented in random order.

The second fMRI task was an aversive extinction task (EV) which was scanned 45 min after acquisition in the first MID task. Here, the aversive cue, anticipation phase and target were shown followed by grey images only with the same timings as the MID task. This task consisted of 50 trials.

### Imaging acquisition and processing

Acquisition parameters for imaging and preprocessing steps are described in the Supplement. We applied voxel-based and surface-based morphometry (VBM/SBM) to detect whole-brain voxel-wise grey matter volume (GMV) and cortical thickness (CT) differences between HC, OCDc and CAP using the Computational Anatomy Toolbox 12 (CAT12; http://dbm.neuro.uni-jena.de/cat.html), controlling for age, gender, years of education and/or total intracranial volume (TIV). We further performed region-based morphometry using 126 regions of interest (ROIs) from the Neuromorphometric atlas (provided by Neuromorphometrics, Inc.; http://neuromorphometrics.com) excluding cerebellar, WM and ventricle areas. Group differences in ROIs were evaluated using one-way ANOVA, followed by FDR correction (*p* < 0.05) for multiple comparisons. Potential associations between whole-brain structural organizations and clinical characteristics in terms of symptom severity were then explored. The analysis of CT index was conducted similar to the GMV.

fMRI task images were preprocessed using the recommended default pipeline within the CONN Functional Connectivity Toolbox [37]. After preprocessing, they were fit with regressors encoding different conditions during the anticipation and feedback phase for the AV and EV task separately and head motion parameters. We used whole-brain voxel-wise analyses with a mixed-effects ANOVA during Anticipation (AV_aversive/AV_neutral/EV_aversive) and Feedback (AV_aversive/AV_neutral/AV_grey/EV_grey) phase to test Group by Anticipation/Feedback type interactions (voxel threshold *p* < 0.005 with cluster threshold *p* < 0.05, FDR correction). We also used a hypothesis driven ROI-based analysis focused on the NAc (generated using WFU Pickatlas (Winston-Salem, North Carolina)). Mean contrast values of the significant interaction were extracted using MarsBaR (http://marsbar.sourceforge.net). Regional structural measures (GMV) were controlled as covariates if activated areas overlapped with structures showing group differences in GMV. Anatomical labelling of significant clusters and local maxima were based on the Harvard-Oxford cortical/subcortical atlases and the automated anatomical atlas 3 (AAL3) [38].

To assess the treatment effects on regional BOLD activity and task-related brain functional connectivity (FC) changes after surgery, mediation analyses and generalized psychophysiological interaction (gPPI) were also performed (see Supplementary Information for further details).

## Results

### Clinical and neuropsychological assessments

Post-surgery CAP (*n* = 27), HC (*n* = 34) and OCDc (*n* = 33) patients had similar demographics and duration of illness (Table 1). CAP subjects relative to OCDc had lower scores in Y-BOCS, along with better quality of life (Q-LES-Q-SF) and daily functioning (SDS). There were no significant differences between post-surgery CAP and OCDc in anxiety (HAMA) or depression (HAMD). In a subgroup of CAP with pre-surgery clinical scores (*n* = 15), pre/post improvements were found in Y-BOCS and depression (HAMD), with a trend in anxiety improvement (HAMA, *p* = 0.06).

**Table 1:** Participant Demographics and Clinical Characteristics.

Characteristics	CAP (mean ± SD)					OCDc (mean ± SD)	HC (mean ± SD)	Statistics	*p*	Effect size
Age, years	32.93 ± 6.56					31.48 ± 8.64	35.09 ± 8.60	1.68	0.19	-
Gender (M/F)	18/9 (*N* = 27)					21/12 (*N* = 33)	19/15 (*N* = 34)	0.82	0.66	-
Age of onset, years	18.56 ± 5.24					19.12 ± 5.59	-	421.00	0.72	-
Duration, years	13.93 ± 6.02					12.47 ± 8.18	-	524.00	0.25	-
Education, years	12.96 ± 3.14					13.91 ± 3.63	14.06 ± 3.41	3.26	0.20	-
Time after surgery, months	49.33 ± 37.27					-	-	-	-	-
SSRIs and/or Anti-psychotics, (Y/N)	16/11					24/9	-	1.21	0.42	-
	**Pre** (*n* = 15)	**Post** (*n* = 27)	**Statistics**	***p***	**Effect size**				(Compared with Post scores in the CAP group)	
YBOCS-total	29.47 ± 8.53	11.78 ± 9.63	5.73	<0.001	1.40	23.00 ± 8.76	—	−4.68	<0.001	1.22
-Obsession	16.73 ± 2.69	6.56 ± 5.50	6.48	<0.001	1.58	13.03 ± 5.12	—	−4.68	<0.001	1.22
-Compulsion	12.73 ± 7.23	5.22 ± 5.21	3.97	0.001	0.97	9.97 ± 6.02	—	−3.28	0.002	0.84
HAMA	20.62 ± 10.95	12.35 ± 8.96	2.03	0.06	–	15.45 ± 8.43	—	−1.36	0.18	—
HAMD	16.62 ± 6.68	10.15 ± 7.69	2.56	0.02	0.67	13.82 ± 8.54	—	−1.73	0.09	—
BDI	16.11 ± 12.07					26.76 ± 16.74	4.82 ± 4.59	33.90	<0.001	0.42
SDS	12.19 ± 9.88					21.32 ± 9.40	—	−3.51	<0.001	0.95
Q-LES-Q-SF	41.37 ± 11.11					34.52 ± 10.04	—	2.38	0.02	0.65

The effect size was presented for significant results and was determined by Cohen’s d for independent/paired *t* tests or partial eta squared for analysis of variance (ANOVA) tests.

*SSRIs* Selective serotonin reuptake inhibitors, *Y-BOCS* Yale–Brown Obsessive-Compulsive Scale, *HAMA* Hamilton Anxiety Rating Scale, *HAMD* Hamilton Depression Rating Scale (17-item version), *BDI* Beck Depression Inventory, *SDS* Sheehan Disability Scale, *Q-LES-Q-SF* Quality of Life Enjoyment and Satisfaction Questionnaire - Short Form, *CAP* obsessive-compulsive disorder *(OCD)* capsulotomy patients, *HC* healthy controls, *OCDc* OCD controls.

There were no group differences in cognitive tasks outcomes that survived correction following multiple comparisons. Trend group differences were found in response inhibition (SST), delayed memory (PRM) and set shifting (IED) (Table 2). We show post-hoc analysis outcomes demonstrating impaired response inhibition in the stop signal reaction time in CAP versus OCDc (*p* = 0.03), impaired delayed recognition memory in CAP versus HC (*p* = 0.007) and set shifting impairments in both CAP (*p* = 0.03) and OCDc (*p* = 0.03) versus HC (Fig. S2). Including demographic and behavioural outcome scores as covariates of no interest to understand the impact on cognitive function showed impaired delayed recognition memory (ANCOVA for three groups: F_2,81_ = 4.45, *p* = 0.01, age, gender, education and BDI as covariates; post hoc CAP versus HC: *p* = 0.02) and response inhibition (ANCOVA for CAP and OCDc group: F_1,45_ = 5.11, *p* = 0.03, age, gender, education, BDI and Y-BOCS as covariates), with set shifting no longer significantly different (ANCOVA for three groups, F_2,77_ = 1.75, *p* = 0.18) (See Table S1 in the Supplementary Information).

**Table 2:** Performances of CANTAB tests in CAP, HC and OCDc group.

Task	CAP (mean ± SD)	HC (mean ± SD)	OCDc (mean ± SD)	ANOVA			post-hoc		
				Statistic	*p*	Effect size	CAP vs OCDc	CAP vs HC	OCDc vs HC
							*p*	*p*	*p*
IED, Intra-Extra Dimensional Set Shifting									
-EEDS^a^	11.22 ± 11.40	4.65 ± 6.04	10.62 ± 10.07	8.35	0.02	0.09	0.94	0.03	0.03
PAL, Paired Associative Learning									
-TEA^a^	22.15 ± 15.07	13.82 ± 12.05	19.03 ± 14.51	5.38	0.07	—	—	—	—
PRM, Pattern Recognition Memory									
-PCD^b,c^	0.77 ± 0.16	0.88 ± 0.12	0.82 ± 0.18	4.83	0.01	0.10	0.21	0.007	0.35
-PCI ^a,c^	0.90 ± 0.10	0.92 ± 0.09	0.91 ± 0.08	1.27	0.53	—	—	—	—
RVP, Rapid Visual Information Processing									
-A^b,c^	0.98 ± 0.02	0.99 ± 0.01	0.98 ± 0.02	2.21	0.12	—	—	—	—
-PFA^a,c^	0.01 ± 0.02	0.00 ± 0.00	0.00 ± 0.02	1.00	0.61	—	—	—	—
SOC, Stockings of Cambridge									
-MNM5^a^	7.59 ± 1.86	6.49 ± 1.28	6.84 ± 1.76	5.06	0.08	—	—	—	—
SST, Stop Signal Task									
-SSRT ^b^	276.02 ± 54.06	255.73 ± 36.38	243.33 ± 51.45	3.33	0.04	0.08	0.03	0.23	0.56
SWM, Spatial Working Memory									
-BE^a^	11.96 ± 9.50	7.44 ± 7.81	9.91 ± 7.61	4.64	0.10	—	—	—	—
-S^a^	7.76 ± 2.55	7.18 ± 2.71	7.94 ± 1.48	1.25	0.54	—	—	—	—

^a^Kruskal-Wallis test followed by Dunn’s test for pairwise comparisons if p_ANOVA_ < 0.05.

^b^One-way ANOVA test followed by Tukey’s test for pairwise comparisons if p_ANOVA_ < 0.05.

^c^Arcsine transformed.

The effect size was determined by partial eta squared.

*IED* intra-extra dimensional set shifting: EEDS the number of incorrect responses across ID/ED.

*PAL* paired associative learning: TEA, the number of times the subject chose the incorrect box for a stimulus on assessment problems (adjusted).

*PRM* pattern recognition memory: PCD, the precent correct patterns selected in the delayed forced-choice condition, PCI, the percent correct patterns selected in the immediate forced-choice condition.

*RVP* rapid visual information processing: A, the signal detection measure of a subject’s sensitivity to the target sequence representing how good the subject is at detecting target sequences; PFA, the number of sequence presentations that were false alarms divided by the number of sequence presentations that were false alarms plus the number of sequence presentations that were correct rejections.

*SOC* stockings of cambridge: MNM5, the mean number of attempts required before obtaining the correct solution in 5 moves problem.

*SST* stop signal task: SSRT, the estimate of time where an individual can successfully inhibit their responses 50% of the time.

*SWM* Spatial Working Memory: BE, the number of incorrect visits to the box in which a token has previously been found; S, the number of times a subject begins a new search pattern different from the same box they started with previously, with lower scores implicating a planned strategy.

*CAP* obsessive-compulsive disorder *(OCD)* capsulotomy patients, *HC* healthy controls, *OCDc* OCD controls.

### Volumetric differences

There were no group differences in global brain parameters (See Table S2 in the Supplementary Information). GMV values extracted from ROIs confirmed 7 regions with significant group differences (one-way ANOVA, *p* < 0.05 FDR correction for multiple comparisons): CAP showed lower GMV in bilateral caudate, thalamus, nucleus accumbens (NAc, treated as covariates in the following task-based fMRI analyses), and right basal forebrain compared to HC and OCDc. OCDc showed higher GMV in left caudate compared to HC and CAP. There were no significant group differences in cortical surface measure (cortical thickness) after correction for multiple comparisons. No correlation was found between symptom severity and structural measures.

### Anticipation phase

The fMRI task performance for accuracy and reaction time did not show significant group differences across different valences (Fig. S3A, B), nor did the number of aversive or grey images presented during the AV task (Fig. S3C).

**Fig. 2:**
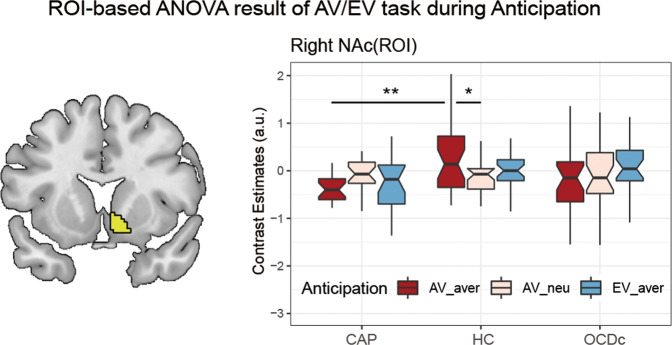
fMRI task results during anticipation phase. Region of interest (right nucleus accumbens, R NAc) analyses showed significant Group (CAP/HC/OCDc) by Anticipation type (AV_aver/AV_neu/EV_aver) interaction. Mean NAc activity extracted from the regions of interest showed decreased NAc activity in CAP during aversive anticipation (AV_aver) relative to HC, and lower activity in response to neutral (AV_neu) compared to aversive anticipation (AV_aver) in HC. CAP obsessive-compulsive disorder (OCD) capsulotomy patients, HC healthy controls, OCDc OCD controls, AV aversive avoidance task, EV aversive extinction task. *p < 0.05, **p < 0.01.

Whole-brain analyses of Group (CAP/HC/OCDc) by Anticipation type (AV_aver/AV_neu/EV_aver) interaction revealed two major clusters of significance (*p*_voxel_ < 0.005, *p*_cluster_ < 0.05 FDR correction): left inferior temporal gyrus (ITG) and right precuneus (Fig. S4 and Table S3). We focused on the hypothesis-driven ROI-based analysis for the NAc [25–27] (controlled for changes in NAc GMV) which demonstrated a significant Group by Anticipation type interaction (F_4,230_ = 3.93, *p* = 0.004) in the right NAc (Fig. 2). Subsequent post hoc tests showed lower activity during aversive anticipation in CAP compared to HC (*p* = 0.002) and a within group hyperactivity in HC for aversive versus neutral anticipation (*p* = 0.01).

### Feedback phase

**Fig. 3:**
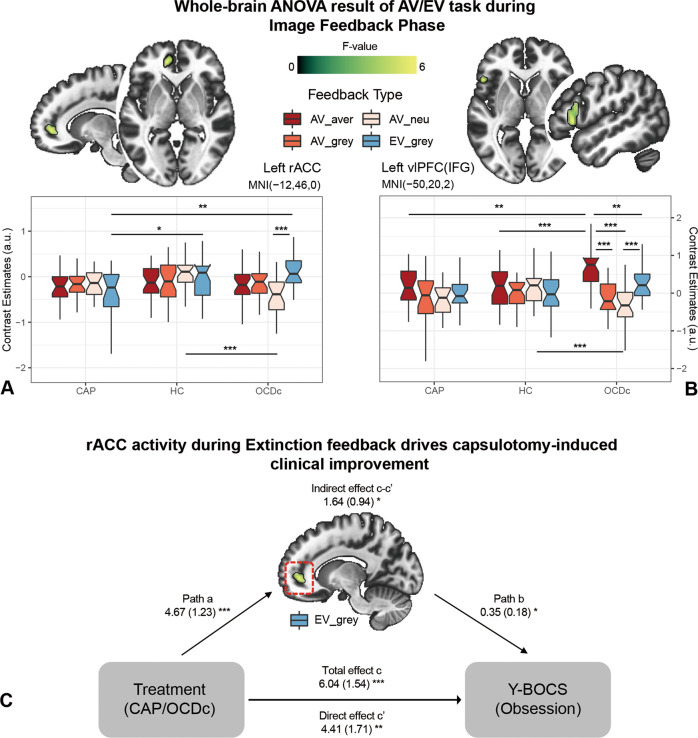
fMRI task results during feedback phase. **A** Left rostral anterior cingulate cortex (rACC), and **B** left ventrolateral prefrontal cortex (vlPFC) showed significant Group (CAP/HC/OCDc) by Feedback type (AV_aver/AV_grey/AV_neu/EV_grey) interaction (*p*_voxel_ < 0.005, *p*_cluster_ < 0.05 FDR correction). **C** Path diagram of the mediation model testing the relationship amongst Treatment (with or without capsulotomy, OCDc or CAP), rACC activity under the condition presenting grey feedback images during the EV task (EV_grey), and symptom severity in obsession (Y-BOCS obsession subscale). Path coefficients are shown next to the arrows with SEs in parentheses. CAP obsessive-compulsive disorder (OCD) capsulotomy patients, HC healthy controls, OCDc OCD controls, AV aversive avoidance task, EV aversive extinction task, IFG inferior frontal gyrus, Y-BOCS Yale–Brown Obsessive-Compulsive Scale. The coordinates are in Montreal Neurological Institute (MNI) space. *p < 0.05, **p < 0.01, ***p < 0.001.

Whole-brain analyses of Group (CAP/HC/OCDc) by Feedback type (AV_aver/AV_grey/AV_neu/EV_grey) interaction showed two major clusters of significance (*p*_voxel_ < 0.005, *p*_cluster_ < 0.05 FDR correction): left rostral anterior cingulate cortex (rACC), and left ventral lateral prefrontal cortex (vlPFC; located within the pars triangularis of the inferior frontal gyrus) (Fig. 3 and Table S4). Post hoc analyses between groups showed OCDc had greater left vlPFC activity to aversive images relative to CAP and HC (Fig. 3B). In contrast, CAP patients showed lower rACC activity during the Extinction feedback (when subjects expected a negative image but observed a grey image) relative to OCDc and HC (Fig. 3A). OCDc also showed lower left vlPFC and rACC activity to neutral images relative to HC. Specifically, within OCDc, greater left vlPFC and rACC activity was observed during Extinction feedback compared to the other conditions with no within-subject differences in the other groups (Fig. 3A, B).

Moreover, we found significant mediation via BOLD activity in rACC during Extinction feedback (coefficient 1.64, SE 0.94, 95% CI 0.08–3.38, *p* < 0.05 bootstrapped) between treatment (with or without capsulotomy, i.e., CAP or OCDc) and clinical response with the Y-BOCS obsession subscale (Fig. 3C) but not with the compulsion subscale or total score. Thus, the effect of capsulotomy on decreasing obsessive symptoms appears to be mediated by downregulation of rACC during Extinction feedback.

### Capsulotomy effects on functional connectivity

**Fig. 4:**
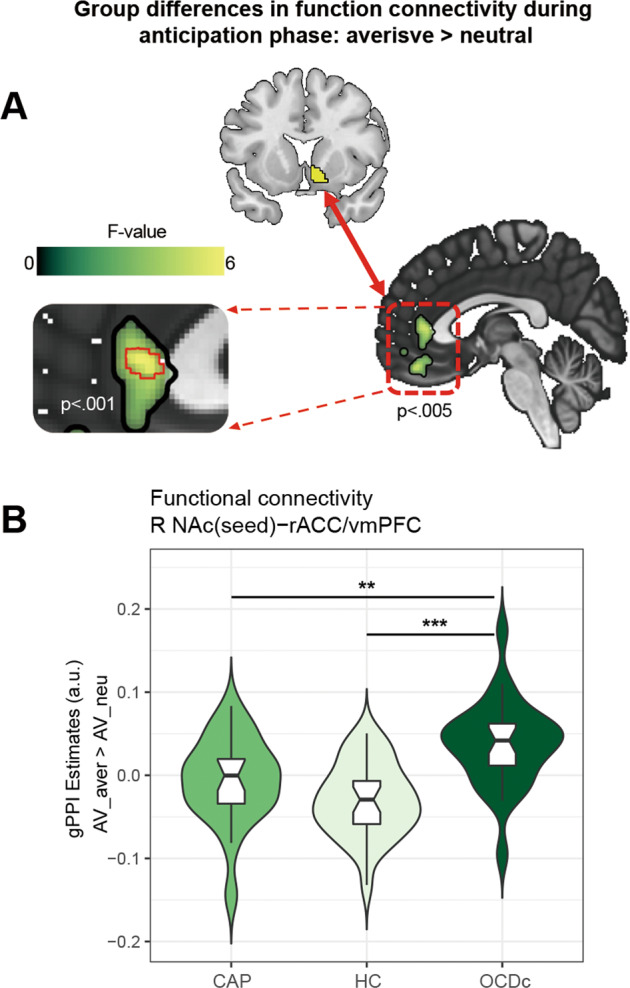
Group differences in functional connectivity measured by generalized psychophysiological interaction (gPPI) contrasting aversive versus neutral anticipation in the AV task (AV_aver>AV_neu). **A** Sagittal slice showing disease and treatment effects on the functional coupling of right NAc (seed) —rACC / vmPFC. Red-outlined squares on the left indicated significance at *p*_voxel_ < 0.001, *p*_cluster_ < 0.05 FDR correction. **B** Mean right NAc – rACC / vmPFC connectivity (gPPI estimate) extracted from regional cluster of significance (*p*_voxel_ < 0.005, *p*_cluster_ < 0.05 FDR correction) revealed significantly decreased functional connectivity in patients after capsulotomy compared to OCD controls contrasting aversive versus neutral anticipation (AV_aver>AV_neu). CAP obsessive-compulsive disorder (OCD) capsulotomy patients, HC healthy controls, OCDc OCD controls, AV aversive avoidance task, EV aversive extinction task, NAc nucleus accumbens, rACC rostral anterior cingulate cortex, vmPFC ventromedial prefrontal cortex. **p < 0.01, ***p < 0.001.

To further elucidate how capsulotomy might influence functional connectivity (FC), we conducted voxel-wise generalized psychophysiological interaction (gPPI) analysis contrasting aversive versus neutral anticipation. We assessed bilateral NAc as seed regions using a whole brain analysis. The right NAc showed significantly greater functional coupling with rACC/vmPFC (Fig. 4) and posterior middle temporal gyrus (Fig. S5A) in OCDc compared to both CAP and HC in the contrast of aversive versus neutral anticipation. We conducted two additional analyses observing aversive and neutral anticipation separately showing similar FC differences in the NAc-rACC/vmPFC coupling under both conditions (Fig. S5F, C). Group differences in FC during aversive trials were also found between NAc and the right vlPFC/IFG, with post hoc analyses showing lower FC in HC compared to both CAP and OCDc (Fig. S5E). During neutral trials, OCDc showed higher FC in the right NAc-posterior cingulate coupling compared to CAP and HC (Fig. S5B). Full results for these contrasts are provided in Table S5. No significant result was found in the left NAc or during the image feedback phase.

## Discussion

**Fig. 5:**
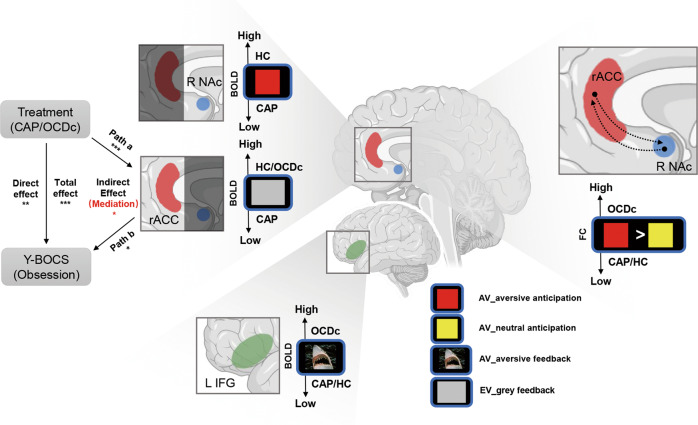
Overview of main findings during aversive avoidance (AV) and aversive extinction task (EV). Significant mediation effect was found via BOLD activity in rACC during Extinction feedback between treatment (with or without capsulotomy, i.e., CAP or OCDc) and clinical response with the Y-BOCS obsession subscale. BOLD Blood oxygen level dependent signal, FC functional connectivity, R NAc right nucleus accumbens, rACC rostral anterior cingulate cortex, L IFG left inferior frontal gyrus. *p < 0.05, **p < 0.01, ***p < 0.001. Note: Example for negative and neutral pictures is taken from the International Affective Picture System (IAPS).

We focused on functional mechanisms underlying capsulotomy for OCD highlighting several novel findings. We confirm a significant improvement in OCD severity six months following capsulotomy and show an important clinically relevant functional improvement in quality of life and disability. Critically we highlight changes following capsulotomy in neural substrates underlying negative affective processing implicating the rostral cingulate cortex, left inferior frontal cortex and nucleus accumbens (Fig. 5). OCD patients showed excessive functional connectivity between the rostral cingulate and nucleus accumbens to the anticipation of aversive relative to neutral imagery which normalized following capsulotomy. Notably these prefrontal regions overlap with the prefrontal corticothalamic tract target identified for optimal clinically effective ALIC or STN DBS for OCD [10, 39, 40]. Thus, our findings following anterior capsulotomy show convergence of tracts highlighting potential mechanisms across both capsulotomy and DBS for OCD implicating particularly the rostral cingulate and negative affective processing. We further investigated cognitive tasks known to be impaired in OCD and mapping across prefrontal substrates and show no significant group differences. We also assessed fMRI tasks implicating other prefrontal regions including working memory (dorsolateral prefrontal cortex), extra-dimensional set shifting (ventrolateral prefrontal cortex) and uncertainty and conflict (dorsal cingulate) and did not show any fMRI differences in the critical comparison of post-capsulotomy and OCD controls (data reported elsewhere). Thus, our findings highlight the clinical efficacy of capsulotomy, its relative safety profile and plausible mechanistic insights that might link DBS and capsulotomy along with potential mechanistic approaches for target optimization with novel ablative procedures. Our approach is predicated on the dimensional RDoC concept that cognitive processes are associated with specific anatomical substrates that might shed light not just on mechanisms but might help guide which fronto-striatal connections might be most functionally relevant given the heterogeneity of OCD subtypes and individual anatomy and extend beyond an anatomical tractography approach. For example, understanding the weighting of negative emotion processing implicating this rostral cingulate region relative to behavioural inflexibility or executive impairment which might implicate ventral prefrontal or dorsolateral prefrontal cortex within each subject or as a function of OCD subtype might be critical in asking which prefrontal-white matter tracts to target.

A comparison review of DBS and capsulotomy outcomes for OCD have suggested that clinical improvements may be superior for capsulotomy [4]. Beyond a symptomatic improvement in obsessive-compulsive symptoms, we further highlight an improvement in clinically relevant outcomes of function and quality of life. Notably capsulotomy does not require extensive clinical follow-up nor risk of neurosurgical complications as DBS. Ablative procedures in principle are more irreversible than DBS but novel technical improvements in ablative approaches such as MRgFUS provide superior precision targeting.

There are several key theoretical mechanistic cognitive constructs underlying OCD that are relevant to neurosurgical targeting [41]. Here we focus on the role of negative emotional processing which might include responses to broader negative emotions [42], or more specific to categories such as disgust [43] or OCD specific content [44]. Potential preclinical models of abnormalities in fear anticipation and fear extinction in OCD have also been proposed [15]. Abnormalities in negative emotional processing and subsequent abnormalities in adaptive flexible behaviours linked to aversive avoidance might also play a critical role underlying OCD [23, 45].

### OCD controls: task validation

We first show task validation in OCD patients and highlight particularly OCD specific responses to uncertain aversive affective outcomes and extinction. Subjects first learn to predict an uncertain negative affective image based on the rapidity and correctness of an instrumental response. Notably the task is very simple involving limited instrumental learning and assesses also aversive Pavlovian conditioning processes. We then investigated a within-session aversive extinction process following acquisition in which an uncertain aversive outcome is expected but instead, no outcome is delivered. In the feedback phase, whole brain analyses identified Group by Feedback type interaction effects in the rostral cingulate and left IFG. In response to aversive affective outcomes, OCD controls showed within-subject hyperactivity of left IFG relative to all other outcomes and also compared to HC, highlighting excessive reactivity and saliency to an uncertain negative affective outcome. In contrast, in response to neutral outcomes, OCD controls showed lower left IFG and rACC activity relative to HC, thus highlighting the differential response to aversive versus neutral stimuli in OCD controls. In the extinction phase, OCD subjects relative to HC show greater activity in rACC to the extinguished outcome. Critically, during the anticipation of aversive versus neutral outcomes during acquisition, OCD controls show greater functional connectivity between rACC and nucleus accumbens compared to healthy controls. Our functional connectivity findings are consistent with observations of aversive conditioning implicating dorsal cingulate hyperactivity in healthy controls [31, 46]. Across paradigms of both emotional and symptom provocation, OCD shows increased activation in the rostral ACC and other areas such as vmPFC, OFC, putamen and amygdala [47]. Together these findings are consistent with abnormalities in hyperactivity and functional connectivity in aversive affective processing identified in OCD [42, 47, 48], and abnormal processing within the corticostriatal loops [50–54]. OCD subjects have also shown impaired extinction learning, a process believed to involve a parallel inhibitory learning process [15]. Similarly, our findings of rACC hyperactivity during the aversive extinguished outcome in OCD may be consistent with maladaptive activity during aversive extinction that might hamper the process of inhibitory extinction learning [15].

### Capsulotomy effects

Critically, post-capsulotomy OCD patients then showed a between-group decrease in hyperactivity to aversive outcomes in the left IFG compared to OCD controls. The left IFG or vlPFC is implicated in multiple functions including inhibitory processes [49], set shifting and switching between goal-directed and habit control [50, 51]. The IFG is also implicated as a marker for saliency with prominent connectivity with amygdala [52], thus consistent with our findings of IFG hyperactivity to aversive stimuli in OCD controls. In response to aversive extinction outcomes, post-capsulotomy patients relative to both OCD and healthy controls showed lower rACC activity with this activity shown to mediate the effects of capsulotomy and OCD obsessional severity changes. Thus, capsulotomy appears to reverse the excessive reactivity to uncertain aversive outcomes in OCD and normalize rACC activity that might be relevant to the learning of safety signals or extinction processes.

We used a ROI-based analysis for the NAc based on previous MID fMRI meta-analyses [31] and further controlled for NAc-specific changes in volume post-capsulotomy. In the anticipation phase, healthy controls showed greater right NAc activity to aversive relative to neutral stimuli. In contrast, capsulotomy subjects showed lower NAc activity to aversive anticipation compared to healthy controls, which is consistent with remediation of excessive reactivity to uncertain aversive stimuli. In the outcome phase, capsulotomy subjects similarly showed bilateral lower NAc activity to positive prediction error (expected aversive outcome but no outcome) during initial learning of the MID task (Fig. S6). This finding parallels with the current understanding of the role of the NAc in discriminating positive/negative valence outcomes and their predictive cues in rodent studies [53–55], and in human imaging studies using fear conditioning paradigms and validated with consistent findings in meta-analyses of fMRI studies [31, 56].

Functional connectivity analysis using gPPI demonstrated hyperactivity in rACC and NAc to aversive relative to neutral anticipation in OCD controls relative to heathy controls, and critically, decreases in connectivity between rACC and NAc in post-capsulotomy patients [57–60]. This change in post-capsulotomy connectivity was observed between rACC and NAc but not with IFG and NAc. Similarly, using a mediation analysis, capsulotomy appears to mediate the effects of rACC activity on Y-BOCS obsession scores. Thus, the tracts between rACC and right NAc appear to be critical to the effects of capsulotomy on obsessional symptoms. On a network level, converging evidence from resting-state and task-based imaging studies suggest abnormal hyperconnectivity along the cortico-striatal axis in OCD [57–60]. Capsulotomy has been shown to decrease resting state functional connectivity between the dorsal cingulate and ventral striatum in OCD subjects [34]. Here we show the effect of capsulotomy on fronto-striatal connectivity across both aversive and neutral imagery consistent with the observation in resting state but critically emphasize a greater decrease in connectivity as a function of affective condition to aversive relative to neutral anticipation. Both non-invasive (e.g., transcranial magnetic stimulation, TMS) or invasive (e.g., deep brain stimulation, DBS) neuromodulations appear to downregulate the mutual communication within this fronto-striatal network [34, 61–63]. Complementing previous findings under rest, here we highlight a valence-dependent interventional capsulotomy effect highlighting a potential underlying mechanistic effect [34, 61–63].

Our findings implicating rostral cingulate and nucleus accumbens activity and connectivity in aversive anticipation and extinction in capsulotomy overlap anatomically with the optimal white matter tract identified for clinical efficacy across DBS targets for OCD. Impairments in aversive conditioning and extinction are hypothesized to underlie the mechanism of action of exposure response prevention, an effective psychotherapeutic intervention [13–16]. We caution that our study is not a pure study of extinction processes as it is tested within session after a 45 min delay, has a simple instrumental component and is also ~60% contingency, hence introducing an element of uncertainty. Our behavioural findings were also limited and thus do not directly test the effects of behavioural measures of extinction learning. Furthermore, there were no differences in the anticipation phase of ‘extinction’ testing but only in the outcome phase. Our findings suggest that the rostral cingulate and its connectivity to the nucleus accumbens are hyperactive in OCD, consistent with impairments in aversive processing and conditioning. This hyperactivity might potentially interfere with the capacity for the parallel inhibitory learning involved in extinction learning. One might speculate that in psychological therapy, rather than avoidance of OCD stimuli, the ongoing exposure might allow for either the parallel inhibitory learning process in extinction or memory reconsolidation and thus increase the capacity to tolerate OCD stimuli and negative affective states. This may similarly be potentially mediated by decreasing rostral cingulate hyperactivity and its connectivity. Thus, our findings indicate a plausible mechanism underlying effective psychotherapeutic intervention, which appears to link across ablative lesions in the ALIC and DBS targets in OCD thus providing a mechanistic link across therapeutic modalities.

We further investigated other cognitive measures known to be impaired in OCD and show no significant group difference that survived multiple correction. Capsulotomy patients tended to identify fewer patterns correctly in the delayed recognition phase (PRM-PCD) compared to HC which was unchanged adjusting for covariates. However, as the difference is not between CAP and OCD controls and further that OCD controls commonly show impaired memory deficits [64–66] as indicated by the lower mean scores that failed to reach significant difference from HC, we suggest that this finding is likely unrelated to surgical intervention and with a larger sample size, the OCD controls would likely also be significantly impaired relative to HC. During the stop signal task, post-capsulotomy subjects showed greater inhibitory impairments relative to OCD controls with the addition of YBOCS scores as a covariate. This suggests that capsulotomy might play a role in impairing inhibitory processes and is unrelated to either the severity of OCD or the improvement in OCD severity post-capsulotomy. There are several plausible explanations: (i) an effect of capsulotomy that might be reflected in the lower left IFG grey matter volume but does not appear to influence the improvement in Y-BOCS scores; or (ii) the OCD control group might be atypical as there were no differences in response inhibition relative to HC as one might expect with severe OCD. If so, the impairment in the CAP group might have been present at baseline and remain unaffected post-capsulotomy. The SSRT impairment might also improve with longer follow-up given observations of cognitive improvements with longitudinal follow-up post-capsulotomy. Our findings also suggest that standard cognitive measures such as set shifting (IFG), working memory (dlPFC), associative learning (vmPFC), and goal-directed planning (dlPFC) commonly impaired in OCD [67] are not remediated nor worsened by capsulotomy. Capsulotomy also does not affect attentional measures (tested using RVP task). These findings highlight the relative safety of the capsulotomy procedure, consistent with findings of no major cognitive effects following capsulotomy particularly with long term follow-up [5, 68–70].

This study is not without limitations. It is cross-sectional, as such we cannot rule out functional and structural differences between OCD control and CAP patients before the surgical intervention. Future studies should include comparisons in brain circuitry involved in emotional processing before and after capsulotomy to investigate the longitudinal impact of capsulotomy. We also acknowledge that a larger sample size is needed to minimize heterogeneity in the patient population. Differential symptom presentations might implicate differential cognitive processes with some studies suggesting impaired aversive processing in contamination fears and greater lateral prefrontal impairments in those with symmetry obsessions. Larger sample sizes might also reveal differences in other cognitive processes that failed to reach significance. We further note that our study focuses on functional connectivity rather than anatomical connectivity. Further studies highlighting the anatomical connection between the prefrontal regions implicated might provide further insight into tracts to target.

Taken together, the present study contributes to the available literature of anterior capsulotomy as an effective and reasonably tolerated treatment option for selected patients with refractory OCD. Our findings also hold potential for novel means of optimizing neuromodulation. These findings extend our current understanding of the therapeutic mechanism of capsulotomy, suggesting a functional modulation in the ventral striatum and rostral cingulate during negative affective processing that unifies DBS and capsulotomy and links with putative mechanisms underlying effective psychotherapeutic interventions.

### Supplementary information

Supplement Material
