# Supplementary material for: Mechanisms underlying capsulotomy for refractory obsessive-compulsive disorder: neural correlates of negative affect processing overlap with deep brain stimulation targets
Source: Mol Psychiatry. 2023 Mar 6;28(7):3063–74. doi: 10.1038/s41380-023-01989-1 (PMC10615758; doi:10.1038/s41380-023-01989-1)
Supplement: Supplementary file 1 — Supplement Material [file 41380_2023_1989_MOESM1_ESM.docx]

**Supplementary Information Appendix for “Mechanisms underlying capsulotomy for refractory obsessive-compulsive disorder: neural correlates of negative affect processing overlaps with deep brain stimulation targets”: SI Materials and Methods, Results, References, Figure Legends, Tables, and Figures**

**SI Materials and Methods**

*Participants and patient selection*

The inclusion and exclusion criteria for patients with obsessive-compulsive disorder (OCD) who underwent capsulotomy (CAP group) have been extensively reported[1-3]. They were considered to be refractory and selected to undergo anterior capsulotomy if they met the following criteria: (i) a primary OCD diagnosis according to the Structured Clinical Interview for DSM-V; (ii) a score on the Yale-Brown Obsessive Compulsive Scale (Y-BOCS) of more than 25 or one subscale score of more than 15; (iii) more than 18 years old, with a course of illness at least 5 years; (iv) non-responsive to or were unable to tolerate at least two trials of selective serotonin reuptake inhibitors of adequate duration and dose; and failed to tolerate augmentation with an atypical antipsychotic; and failed or were unable to tolerate psychotherapy/cognitive behavioural therapy (defined as continuous treatment for more than six months).

The exclusion criteria were as follows: (i) Schizophrenic disorder; bipolar disorder; substance abuse or dependence (except for dependence on nicotine), as assessed with the use of the Mini-International Neuropsychiatric Interview (MINI 6.0.0); (ii) a current severe major depressive episode, determined according to DSM-V criteria (as assessed with the use of the MINI 6.0.0).

OCD control patients (OCDc group) should have a primary diagnosis of OCD according to DSM-V and age over 18. The exclusion criteria are the same with the OCD capsulotomy patients.

Healthy controls (HC group) needed to be (i) over the age of 18; (ii) No obvious physical or mental disorders; and (iii) a Beck Depression Inventory Second Edition (BDI-II) score less than 19.

*Surgical procedure*

The lesion locations for each patient (n=25) are shown in high-resolution T1-weighted images (**Figure S1**). Mean lesion size was 1613.1 mm^3^ (standard deviation = 740.5; range 701.0 – 3912.0 mm^3^). For more detailed information on the surgical procedure please refer to our previously published work [3].

*Description of CANTAB outcome measures*

We focused on primary outcome measures in the Cambridge Neuropsychological Test Automated Battery (CANTAB). As there were no significant findings (corrected for multiple comparisons) in these measures, we have not elaborated on task details. In the Intra-Extra Dimensional Set Shift task (IED), the primary outcome measure was the number of trials with incorrect responses (IED-EEDS). In the Paired Associative Learning task (PAL), the primary outcome measure was the total errors made across trials adjusted for the estimated number of errors they would have made on any problem (PAL-TEA), which enables the direct comparison across all subjects regardless of those who terminated before completing the final stage of the task. In the Pattern Recognition Memory task (PRM), two measures were assessed: (i) percent correct patterns selected in the delayed (PRM-PCD) and (ii) the immediate forced-choice condition (PRM-PCI). In the Rapid Visual Information Processing task (RVP), we analysed (i) the probability of false alarms (RVP-PFA, False Alarms ÷ (False Alarms + Correct Rejections)) and (ii) a signal detection measure of how good the subject is at detecting target sequences (RVP-A). In the Stocking of Cambridge task (SOC), the primary outcome measure was the mean number of attempts required prior to successful resolution in the most difficult 5 move condition (SOC-MNM5). In the Stop Signal task (SST), the primary outcome measure was the stop signal reaction time (SST-SSRT), which provides an index of response inhibition with higher scores being more impaired. In the Spatial Working Memory task (SWM), we analysed (i) the number of incorrect visits to boxes which a token has previously been found (SWM-BE), and (ii) the number of times a subject begins a new search pattern different from the same box they started with previously (SWM-S), with lower score implicating a planned strategy.

*MRI Data Acquisition*

Data were acquired on a 3.0 Tesla Siemens Prism MR scanner equipped with a circular-polarized head coil with a T2*-weighted single-shot gradient echo planar imaging sequence: 33 slices, 2.0 × 2.0 × 3.0 mm resolution, TE = 30 ms, TR = 2000 ms, flip angle = 90°, interleaved acquisition (from bottom to top) for each run. A vacuum head cushion was used to immobilize the participants' heads and necks in order to reduce movement artefacts. Earplugs were provided to attenuate background noise and additional headphones were used to communicate with subjects. Stimuli were generated using Presentation (Neurobehavioral Systems) and were projected by means of a mirror system attached to the head coil. Anatomical high-resolution T1-weighted scans (spatial resolution 0.8 × 0.8 × 0.8 mm, TR = 3000 ms, TE = 2.56 ms, flip angle = 7°) were acquired for further analyses in volumetric measures and brain morphometry.

*Data Processing*

Functional images were preprocessed using the default method within the CONN Functional Connectivity Toolbox [4] for slice timing correction, motion correction (realignment), image normalization and coregistration image resampling at a 2 × 2 × 2 mm^3^ voxel size, and 6-mm Gaussian smoothing. Artifact Detection Tools (ART, http://www.nitrc.org/projects/artifact_detect/) software package was used for automatic detection of outliers with extreme spike and motion (z threshold 6, movement threshold of 3 mm). Subjects were excluded if >20% of scans hit the threshold. Framewise displacement (FD) [5] was then calculated to identify time points with > 0.5 mm motion. No group difference of FD was found in the aversive (F2,78 =0.77, p=0.47, η_p_^2^=0.02) and extinction (F2,78 =0.55, p=0.58, η_p_^2^=0.01) task. After regressing out the six motion parameters during the first-level analysis, mean FD value of each subject included was treated as an additional covariate in the second-level analysis, and no significant difference in brain activation was found compared to non-FD ones. Note that data of 6 OCDc, 3 CAP and 3 HC were not included for subsequent brain-wide analysis due to head motion (larger than 3 mm translation displacement) or missing of time points (failed to finish the task) during MRI scanning, resulting in 27 OCDc, 22 CAP and 31 HC to be included in the further analyses.

Images were then analysed by General Linear Model (GLM) using SPM12 (Wellcome Trust Centre for Neuroimaging, London, UK). Specifically, regressors (task conditions) and six motion parameters were convolved with the canonical hemodynamic response function. A temporal high-pass filter of 128s was applied to the data to eliminate low-frequency noise. The general linear model (GLM) was fit to the aversive avoidance task (AV) and aversive extinction task (EV) task separately, and contrast estimates were combined across each.

To determine whether treatment effects on regional BOLD activity during different phases of AV/EV task critically contributed to the symptom improvement, we employed an exploratory ROIs-based mediation analyses applying clusters showing significant interaction in the GLM analysis as mediators between treatment (with or without capsulotomy, i.e., OCDc or CAP) and clinical outcome (measured using Y-BOCS or its subtotals in obsession and compulsion). Statistical significance was determined using a bootstrapping approach with 10,000 samples.

To assess the task-related brain functional connectivity changes after surgery, generalized psychophysiological interaction (gPPI) [6] was applied to identify the condition-dependent changes of FC between seed regions and the rest of the brain. The seeds of interest includes the nucleus accumbens (NAc, same as the hypothesis driven ROI applied in the fMRI aversive avoidance and aversive extinction task analyses), and brain regions showing significant interaction using whole-brain voxel-wise analyses, defined as 6 mm radius spheres centred around the global maximum coordinate in MNI space [MNI(+12,+46,0)] located within the rostral anterior cingulate cortex (rACC), and [MNI(-50,20,2)] located within the left inferior frontal cortex. Condition-dependent changes (aversive versus neutral anticipation/image feedback contrasts, and aversive/neutral condition alone) in functional connectivity were then assessed using one-way ANOVA/ANCOVA on extracted gPPI estimates with corresponding grey matter volumes of seed regions as covariates if necessary (seed in NAc). Connectivity maps were corrected at a voxel threshold of p<0.005 with cluster p<0.05, FDR correction.

**SI Results**

*fMRI task performance: accuracy and reaction time against target responses during the aversive avoidance task (AV)*

A mixed-effect ANOVA on arcsine-transformed accuracy towards target responses during the AV task revealed no Group (CAP/OCDc/HC) by Anticipation type (AV_aver/AV_neu/EV_aver) interaction effect (F_4,76_ =1.46, p=0.24, η_p_^2^=0.04) or Group main effect (F_2,76_ =1.42, p=0.25, η_p_^2^=0.04). A marginally significant main effect in Anticipation type was found (F_2,76_=3.77, p=0.053, η_p_^2^=0.05), and was driven by a better performance towards neutral compared to aversive trials as the post hoc analyses revealed (see **Fig.S3A**). Age was found to be negatively correlated with accuracy during aversive trials in HC (r_Pearson_ =-0.51, p=0.005) but not in OCDc or CAP. No significant correlation was found between accuracy and years of education, age of onset, Y-BOCS or BDI scores between the three groups across different conditions. We further examined the reaction time (RT, milliseconds) across groups and conditions and find no significant main effect or interaction (F_4,76_ =1.26, p=0.29, η_p_^2^=0.031) (see **Fig.S3B**). Correlation analyses on RT revealed positive relationship with age in HC and OCDc (both r_Pearson_>0.60, p<0.001), indicating a quicker response towards targets among younger subjects in these two groups. In the CAP group, the older the age of onset, the longer the time taken to respond to the target, as RT was positively correlated with age of onset in both aversive (r_Pearson_=0.49, p<0.05) and neutral conditions (r_Pearson_=0.55, p<0.01). No significant correlation with RT was found among other possible demographic and clinical features. No significant group difference was found on the number of aversive/grey pictures presented throughout the AV task (F_2,77_ =1.72, p=0.19, η_p_^2^=0.04).

*Feedback phase: ROI-based analyses*

ROI-based analyses in the bilateral NAc during the Feedback phase showed a Group (CAP/OCDc/HC) by Feedback type (AV_aver/AV_grey/AV_neu/EV_grey) interaction (**Fig.S6**). Post-hoc analyses showed that CAP group had lower bilateral NAc activity relative to HC when observing grey images during the AV task. In HC, within subject contrasts during the AV task revealed lower bilateral NAc activity against aversive compared to grey images with no differences observed in other groups.

*Feedback phase: Mediation outcome*

The mediation models tested between different treatment (with/without capsulotomy) and clinical outcome (Y-BOCS and obsession/compulsion subtotals) confirmed a significant association via rACC activity during the Feedback phase of the EV task (EV_grey). The regression coefficient between treatment and bold activity of rACC during the Extinction feedback conditions (when subjects expected a negative image but observed a grey image) was significant (coefficient 4.67, SE 1.23, p<0.001; **Fig.3C**, path a), which confirms the impact from the independent variable to the mediator in the model. Likewise, a significant association between BOLD activity in the rACC and symptom severity was observed in the model (coefficient 0.35, SE 0.18, p<0.05; **Fig.3C**, path b). Overall, the mediation model revealed that the relationship between treatment (capsulotomy surgery) and outcome (obsessive symptoms assessed by Y-BOCS obsession subscale) is partially mediated by the BOLD activity in rACC during Extinction feedback (coefficient 1.64, SE 0.94, 95%CI 0.08-3.38, p<0.05 (bootstrapped); **Fig.3C**, indirect effect path c-c’=a×b).

**References**

1. Jung HH, Kim SJ, Roh D, Chang JG, Chang WS, Kweon EJ, et al. Bilateral thermal capsulotomy with MR-guided focused ultrasound for patients with treatment-refractory obsessive-compulsive disorder: a proof-of-concept study. Mol Psychiatry. 2015;20(10):1205-11.

2. Lopes AC, Greenberg BD, Canteras MM, Batistuzzo MC, Hoexter MQ, Gentil AF, et al. Gamma ventral capsulotomy for obsessive-compulsive disorder: a randomized clinical trial. JAMA Psychiatry. 2014;71(9):1066-76.

3. Yin D, Zhang C, Lv Q, Chen X, Zeljic K, Gong H, et al. Dissociable Frontostriatal Connectivity: Mechanism and Predictor of the Clinical Efficacy of Capsulotomy in Obsessive-Compulsive Disorder. Biol Psychiatry. 2018;84(12):926-36.

4. Whitfield-Gabrieli S, Nieto-Castanon A. Conn: a functional connectivity toolbox for correlated and anticorrelated brain networks. Brain Connect. 2012;2(3):125-41.

5. Power JD, Mitra A, Laumann TO, Snyder AZ, Schlaggar BL, Petersen SE. Methods to detect, characterize, and remove motion artifact in resting state fMRI. Neuroimage. 2014;84:320-41.

6. McLaren DG, Ries ML, Xu G, Johnson SC. A generalized form of context-dependent psychophysiological interactions (gPPI): a comparison to standard approaches. Neuroimage. 2012;61(4):1277-86.

**SI Figure Legends**

**Supplementary Fig.1.** The lesion locations for each patient presented in high-resolution T1-weighted images. MR images were not acquired from 2 CAP subjects for individual reasons (CAP_010 and CAP_013).

**Supplementary Fig.2.** Boxplots comparing CANTAB task performance in set shifting (A), delayed memory (B), and response inhibition (C), unadjusted for covariates (one-way ANOVA). Scatterplots (D) showed no significant correlation between Y-BOCS scores and SSRT in OCDc (excluding 3 outliers) and CAP group. Abbreviations: CAP, obsessive-compulsive disorder (OCD) capsulotomy patients; HC, healthy controls; OCDc, OCD controls; IED-EEDS, the number of incorrect responses (EEDS) during Intra-Extra Dimensional Set Shifting (IED) task; PRM-PCD, the precent correct patterns (PCD) selected in the delayed forced-choice condition during Pattern Recognition Memory (PRM) task; SSRT, the stop signal reaction time (in milliseconds, ms) measured using the Stop Signal Task (SST). The solid lines are the linear regression with shaded areas depicting the 95% confidence interval. * p<0.05, ** p<0.01.

**Supplementary Fig.3.** Task performances depicted by (A) the accuracy rate against the targets and (B) the response time revealed no significant Group (CAP/HC/OCDc) by Valence (AV_aver/AV_neu/EV_aver) interaction or main effects. (C) The number of aversive or grey pictures presented during aversive trials in three groups (based on the response towards the target arrow following the anticipatory period) revealed no significant interaction or main effects. Abbreviations: CAP, obsessive-compulsive disorder (OCD) capsulotomy patients; HC, healthy controls; OCDc, OCD controls; AV, aversive avoidance task; EV, aversive extinction task.

**Supplementary Fig.4.** (A) Right precuneus and (B) left inferior temporal gyrus (ITG) showing significant Group (CAP/HC/OCDc) by Anticipation type (AV_aver/AV_neu/EV_aver) interaction (p_voxel_ <0.005, p_cluster_ <0.05 FDR correction). Abbreviations: CAP, obsessive-compulsive disorder (OCD) capsulotomy patients; HC, healthy controls; OCDc, OCD controls; AV, aversive avoidance task; EV, aversive extinction task. The coordinates are in Montreal Neurological Institute (MNI) space. * p<0.05, ** p<0.01, *** p<0.001.

**Supplementary Fig.5.** Group differences of functional connectivity (FC) using generalized psychophysiological interaction (gPPI) during the anticipation phase seeded in the right nucleus accumbens (NAc). (A) Axial and sagittal views showing stronger FC between right NAc and left posterior middle temporal gyrus (pMTG) in OCDc compared to HC (p<0.001) and CAP (p=0.02) under the contrast of aversive versus neutral anticipation (AV_aver>AV_neu). (B) Sagittal view showing stronger FC between right NAc and posterior cingulate cortex (PCC) in OCDc compared to both HC (p=0.001) and CAP (p<0.001) during neutral anticipation (AV_neu). (C) Sagittal view showing stronger FC between right NAc and rostral anterior cingulate cortex (rACC)/ventral medial prefrontal cortex (vmPFC) in OCDc compared to both HC (p<0.001) and CAP (p=0.007) during neutral anticipation (AV_neu). (D) Axial and sagittal views showing stronger FC between right NAc and left posterior middle temporal gyrus (pMTG) in both CAP (p=0.01) and OCDc (p=0.002) compared to HC during aversive anticipation (AV_aver). (E) Sagittal view showing stronger FC between right NAc and right inferior frontal gyrus (IFG) in both OCDc (p=0.003) and CAP (p=0.005) relative to HC during aversive anticipation (AV_aver). (F) Sagittal slice showing stronger FC between right NAc and rACC/vmPFC in OCDc compared to HC (p<0.001) during aversive anticipation (AV_aver). Abbreviations: CAP, obsessive-compulsive disorder (OCD) capsulotomy patients; HC, healthy controls; OCDc, OCD controls; AV, aversive avoidance task; EV, aversive extinction task. The images were thresholded at p_voxel_ <0.005, p_cluster_ <0.05 FDR correction. *p<0.05, **p<0.01, ***p<0.001.

**Supplementary Fig.6.** ROI-based (nucleus accumbens, NAc) analyses revealed significant Group (CAP/HC/OCDc) by Feedback type (AV_aver/AV_grey/AV_neu/EV_aver) interaction (p_voxel_ <0.005, p_cluster_ <0.05 FDR correction). (A) Mean left NAc activity extracted from the region of interest showed deactivated NAc activity in HC during aversive versus grey feedback (p=0.03), and lower activity in CAP in response to grey feedbacks compared to HC (p=0.01). (B) Mean right NAc activity extracted from the ROI showed deactivated NAc activity in HC during aversive versus grey feedback (p<0.001), lower activity in CAP in response to grey images during extinction phase (EV_grey) compared to HC (p=0.002) and OCDc (p<0.001), and stronger activity against grey images (AV_grey) in HC compared to CAP (p=0.003). Abbreviations: CAP, obsessive-compulsive disorder (OCD) capsulotomy patients; HC, healthy controls; OCDc, OCD controls; AV, aversive avoidance task; EV, aversive extinction task. *p<0.05, **p<0.01, ***p<0.001.

**SI Tables**

**Table S1.** Performances of CANTAB tests in CAP, HC and OCDc group controlling for demographic and behavioural outcome scores.

| Task | CAP (mean ± SD) | HC (mean ± SD) | OCDc (mean ± SD) | ANCOVA | | | Post hoc | | |
| --- | --- | --- | --- | --- | --- | --- | --- | --- | --- |
|  |  |  |  | **Statistic** | **P** | **Effect size** | **CAP vs OCDc** | **CAP vs HC** | **OCDc vs HC** |
|  |  |  |  |  |  |  | **p** | **p** | **p** |
| IED, Intra-Extra Dimensional Set Shifting | | | | | | | | | |
| -EEDS ^a^ | 11.22±11.40 | 4.65±6.04 | 10.62±10.07 | 1.75 | 0.18 | 0.04 | - | - | - |
| PRM, Pattern Recognition Memory | | | | | | | | | |
| -PCD ^b, d^ | 0.77±0.16 | 0.88±0.12 | 0.82±0.18 | 4.45 | 0.01 | 0.10 | 0.12 | 0.02 | 0.74 |
| SST, Stop Signal Task | | | | | | | | | |
| -SSRT ^c^ | 276.02±54.06 | 255.73±36.38 | 243.33±51.45 | 5.11 | 0.03 | 0.10 | 0.03 | - | - |

^a^ Quade’s test (non-parametric analysis of covariance, ANCOVA) with group (CAP/HC/OCDc) as fixed factor and age, gender, years of education and BDI as covariates.

^b^ One-way ANCOVA test with group (CAP/HC/OCDc) as fixed factor and age, gender, years of education, and BDI as covariates.

^c^ One-way ANCOVA test with group (CAP and OCDc) as fixed factor and age, gender, years of education, BDI and Y-BOCS as covariates (Y-BOCS was added here as a covariate as post hoc results of one-way ANOVA suggesting group difference in SSRT was between CAP and OCDc).

^d^ Arcsine transformed.

Abbreviations: EEDS, the number of incorrect responses across intra-extra dimensions; PCD, the precent correct patterns selected in the delayed forced-choice condition; SSRT, the estimate of time where an individual can successfully inhibit their responses 50% of the time; BDI, Beck Depression Inventory; Y-BOCS, Yale-Brown Obsessive Compulsive Scale; CAP, obsessive-compulsive disorder (OCD) capsulotomy patients; HC, healthy controls; OCDc, OCD controls. The effect size was determined by partial eta squared.

**Table S2.** Group differences for grey matter volume (GMV) and cortical thickness (CT) between CAP, HC and OCDc groups in predefined volume/surface-based ROIs.

| Region and hemisphere | CAP (N=25) | HC (N=32) | OCDc (N=30) | Statistics | p | Effect size |
| --- | --- | --- | --- | --- | --- | --- |
|  | **GMV; mean (s.d.)** | | |  |  |  |
| Brain parameters (cm^3^) | | | | | | |
| TIV | 1505.10 (139.94) | 1470.37 (169.13) | 1507.72 (134.46) | 0.59 | 0.56 | - |
| GM | 668.05 (63.32) | 668.95 (64.80) | 683.11 (61.65) | 0.52 | 0.60 | - |
| WM | 541.18 (46.70) | 541.81 (72.54) | 549.63 (49.76) | 0.19 | 0.83 | - |
| CSF | 295.88 (55.68) | 259.60 (60.60) | 274.98 (55.24) | 2.74 | 0.07 | - |
| Regions of significance (mm^3^) | | | | | | |
| Thalamus |  |  |  |  |  |  |
| Left | 4306.23 (512.94) | 4947.14 (380.58) | 5157.36 (418.35) | 27.18 | <0.001 | 0.40 |
| Right | 4508.35 (460.36) | 5227.26 (431.97) | 5429.31 (411.23) | 32.46 | <0.001 | 0.44 |
| Putamen |  |  |  |  |  |  |
| Left | 3604.68 (652.40) | 3733.36 (503.18) | 3984.42 (409.55) | 3.80 | 0.03 | 0.08 |
| Right | 3646.28 (508.71) | 3842.05 (457.35) | 4072.42 (424.34) | 5.77 | 0.005 | 0.12 |
| Caudate |  |  |  |  |  |  |
| Left | 2267.86 (467.56) | 2660.38 (247.12) | 2885.14 (357.07) | 20.55 | <0.001 | 0.33 |
| Right | 2560.10 (402.88) | 2832.66 (251.23) | 3036.76 (335.55) | 14.04 | <0.001 | 0.25 |
| NAc |  |  |  |  |  |  |
| Left | 297.83 (44.69) | 386.35 (43.35) | 399.85 (44.98) | 40.55 | <0.001 | 0.49 |
| Right | 333.07 (50.09) | 395.71 (44.09) | 411.63 (45.36) | 20.92 | <0.001 | 0.34 |
| Basal Forebrain |  |  |  |  |  |  |
| Right | 721.81 (72.5) | 784.30 (81.8) | 797.14 (90.84) | 6.16 | 0.003 | 0.13 |
| IFG (orbital part) |  |  |  |  |  |  |
| Left | 1382.86 (163.40) | 1492.55 (186.88) | 1535.14 (189.83) | 4.87 | 0.01 | 0.11 |
| Right | 1390.44 (158.94) | 1411.88 (211.58) | 1518.24 (258.79) | 2.83 | 0.06 | - |
|  | **CT; mean (s.d.)** | | |  |  |  |
| Regions of significance (mm) | | | | | | |
| IFG (pars opercularis) |  |  |  |  |  |  |
| Left | 2.57 (0.10) | 2.64 (0.10) | 2.66 (0.15) | 3.84 | 0.03 | 0.08 |
| Right | 2.57 (0.11) | 2.64 (0.14) | 2.66 (0.15) | 3.36 | 0.04 | 0.07 |
| IFG (pars triangularis) |  |  |  |  |  |  |
| Left | 2.48 (0.11) | 2.58 (0.13) | 2.55 (0.16) | 3.26 | 0.04 | 0.07 |
| OFG (lateral) |  |  |  |  |  |  |
| Left | 2.59 (0.13) | 2.67 (0.11) | 2.67 (0.11) | 5.20 | 0.007 | 0.11 |
| MFG (caudal) |  |  |  |  |  |  |
| Left | 2.55 (0.12) | 2.63 (0.11) | 2.65 (0.16) | 4.14 | 0.02 | 0.09 |
| Right | 2.56 (0.13) | 2.65 (0.12) | 2.69 (0.17) | 5.55 | 0.009 | 0.12 |
| MFG (rostral) |  |  |  |  |  |  |
| Left | 2.37 (0.12) | 2.50 (0.10) | 2.45 (0.14) | 3.55 | 0.03 | 0.08 |
| Right | 2.39 (0.10) | 2.50 (0.10) | 2.47 (0.14) | 4.12 | 0.02 | 0.09 |
| PHG |  |  |  |  |  |  |
| Left | 2.46 (0.19) | 2.38 (0.16) | 2.51 (0.19) | 3.97 | 0.02 | 0.09 |
| Right | 2.45 (0.14) | 2.39 (0.20) | 2.50 (0.15) | 3.68 | 0.03 | 0.08 |
| Temporal Pole |  |  |  |  |  |  |
| Right | 3.28 (0.31) | 3.43 (0.26) | 3.57 (0.29) | 5.90 | 0.004 | 0.12 |

Abbreviations: TIV, Total intracranial volume; GM, Grey matter, WM; White matter; CSF, Cerebrospinal fluid; NAc, Nucleus accumbens; IFG, Inferior frontal gyrus; OFG, Orbitofrontal gyrus; MFG, Middle frontal gyrus; PHG, Parahippocampal gyrus; CAP, obsessive-compulsive disorder (OCD) capsulotomy patients; HC, healthy controls; OCDc, OCD controls. The effect size was determined by partial eta squared. s.d., Standard deviation.

**Table S3.** Brain regions underlying significant whole-brain voxel-wise Group (CAP/HC/OCDc) by Anticipation type (AV_aver/AV_neu/EV_aver) interaction during the aversive avoidance task (AV) and aversive extinction task (EV).

| Brain Regions | L/R | Brodmann area (BA) | Cluster extent (K_E_) | Statistics | Effect size | Peak MNI coordinates  (x, y, z) | | |
| --- | --- | --- | --- | --- | --- | --- | --- | --- |
| Voxel p<0.005, cluster p<0.05, FDR correction | | | | | | | | |
| Inferior Temporal Gyrus (ITG) | L | 20 | 199 | 9.75 | 0.20 | -54 | -28 | -30 |
| Precuneus | R | 31 | 125 | 3.98 | 0.09 | 4 | -44 | 48 |
| Voxel p<0.001, cluster p uncorrected | | | | | | | | |
| Bed Nucleus of the Stria Terminalis (BNST) | L | - | 7 | 5.60 | 0.15 | -6 | 6 | 4 |
| Nucleus Accumbens (NAc) | R | - | 25 | 7.94 | 0.11 | 6 | 12 | -2 |

Abbreviations: CAP, obsessive-compulsive disorder (OCD) capsulotomy patients; HC, healthy controls; OCDc, OCD controls. The effect size was determined by partial eta squared.

**Table S4.** Brain regions underlying significant whole-brain voxel-wise Group (CAP/HC/OCDc) by Feedback type (AV_aver/AV_grey/AV_neu/EV_grey) interaction during the aversive avoidance task (AV) and aversive extinction task (EV).

| Brain Regions | L/R | Brodmann area (BA) | Cluster extent (K_E_) | Statistics | Effect size | Peak MNI coordinates  (x, y, z) | | |
| --- | --- | --- | --- | --- | --- | --- | --- | --- |
| Voxel p<0.005, cluster p<0.05, FDR correction | | | | | | | | |
| Rostral anterior cingulate cortex (rACC) | L | 32 | 98 | 5.61 | 0.13 | -12 | 46 | 0 |
| Inferior frontal gyrus (Pars triangularis) | L | 45 | 139 | 5.90 | 0.12 | -50 | 20 | 2 |

Abbreviations: CAP, obsessive-compulsive disorder (OCD) capsulotomy patients; HC, healthy controls; OCDc, OCD controls. The effect size was determined by partial eta squared.

**Table S5.** Brain regions underlying significant whole-brain voxel-wise between Group (CAP/HC/OCDc) differences in functional connectivity seeded in the right nucleus accumbens (NAc) during the anticipation phase of aversive avoidance task (AV).

| Brain Regions | L/R | Brodmann area (BA) | Cluster extent (K_E_) | Statistics | Effect size | Peak MNI coordinates  (x, y, z) | | |
| --- | --- | --- | --- | --- | --- | --- | --- | --- |
| Voxel p<0.005, cluster p<0.05, FDR correction | | | | | | | | |
| Anticipation phase: Aversive > Neutral | | | | | | | | |
| Rostral anterior cingulate cortex (rACC)/ventral medial prefrontal cortex (vmPFC) | L/R | 31/32 | 405 | 5.25 | 0.17 | 4 | 38 | 6 |
| Middle temporal gyrus, posterior (pMTG) | L | 21 | 252 | 4.87 | 0.17 | -60 | -24 | -8 |
| Anticipation phase: Aversive | | | | | | | | |
| Rostral anterior cingulate cortex (rACC)/ventral medial prefrontal cortex (vmPFC) | L/R | 31/32 | 1178 | 12.29 | 0.24 | 4 | 36 | 6 |
| Middle temporal gyrus, posterior (pMTG) | L | 21 | 262 | 4.20 | 0.16 | -60 | -24 | -6 |
| IFG (Pars triangularis) | R | 45 | 130 | 3.72 | 0.16 | 54 | 32 | 6 |
| Anticipation phase: Neutral | | | | | | | | |
| Rostral anterior cingulate cortex (rACC)/ventral medial prefrontal cortex (vmPFC) | L/R | 31/32 | 384 | 10.81 | 0.22 | 2 | 40 | 6 |
| Posterior cingulate cortex (PCC) | R | 23 | 156 | 9.81 | 0.21 | 8 | -54 | 14 |

Abbreviations: CAP, obsessive-compulsive disorder (OCD) capsulotomy patients; HC, healthy controls; OCDc, OCD controls. The effect size was determined by partial eta squared.

**SI Figures**

**
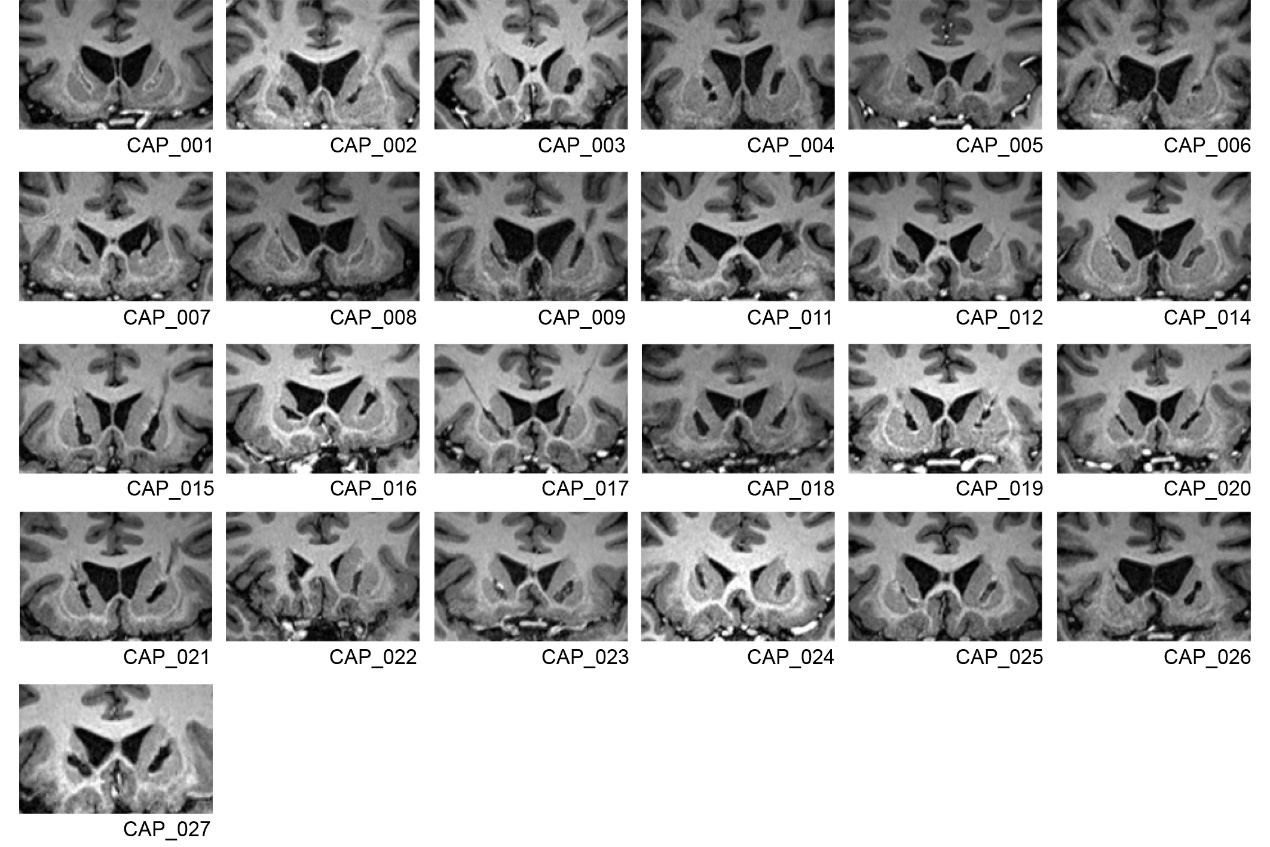
**

Supplementary Fig.1

**
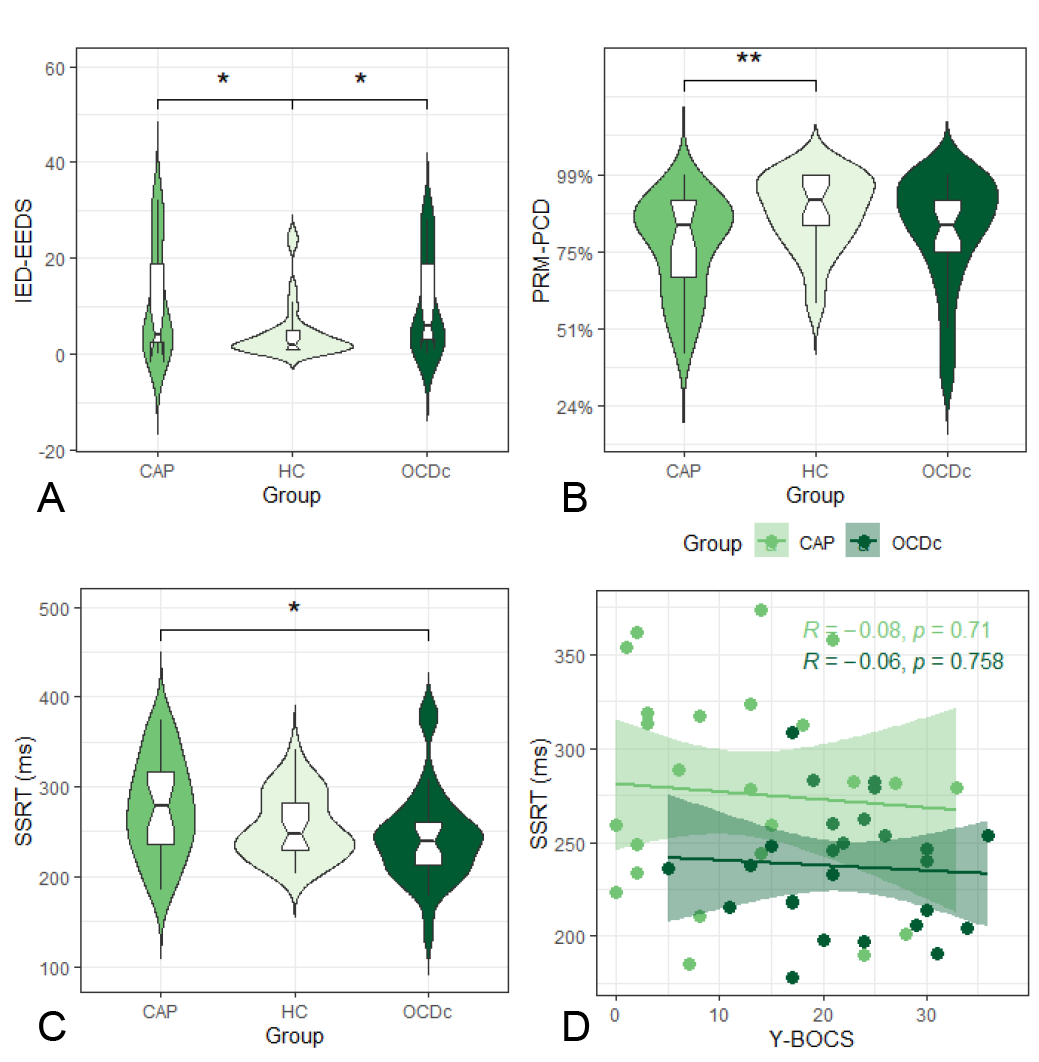
**

Supplementary Fig.2

**
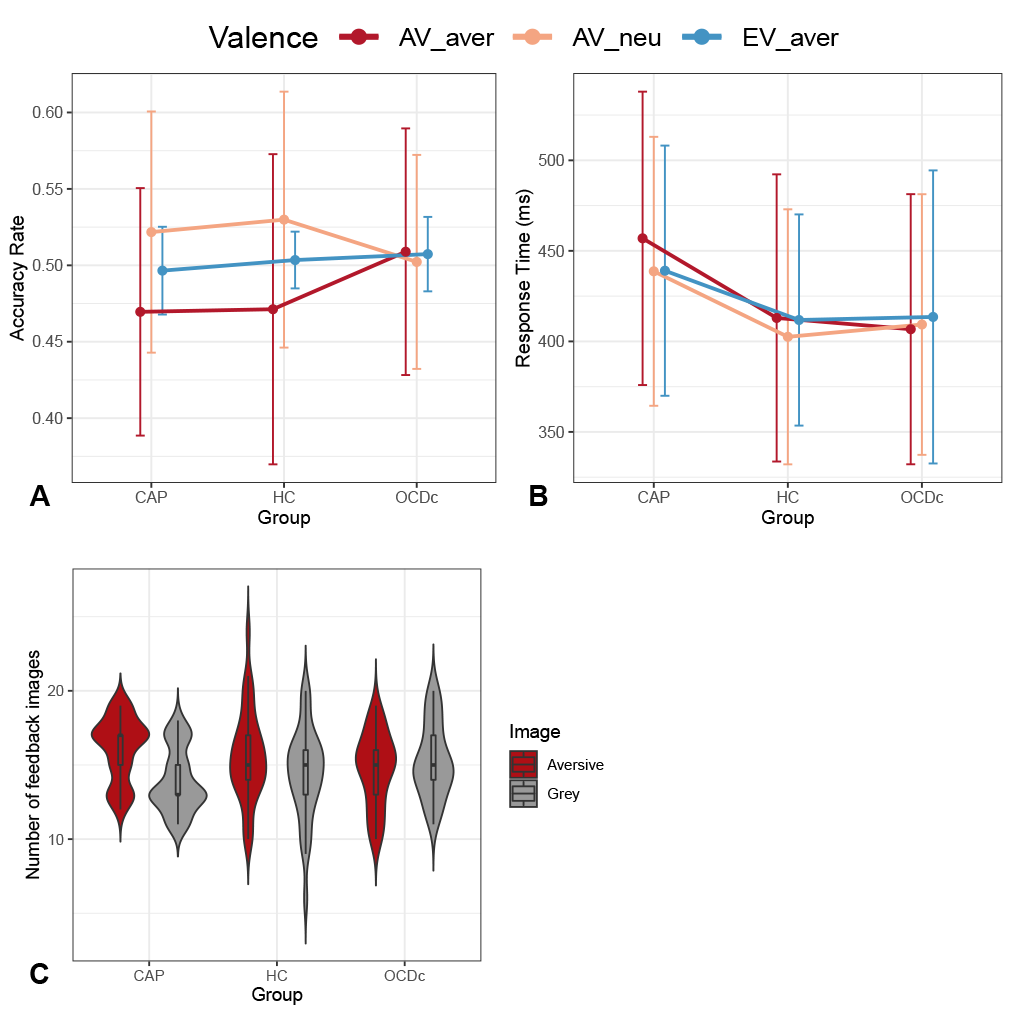
**

Supplementary Fig.3


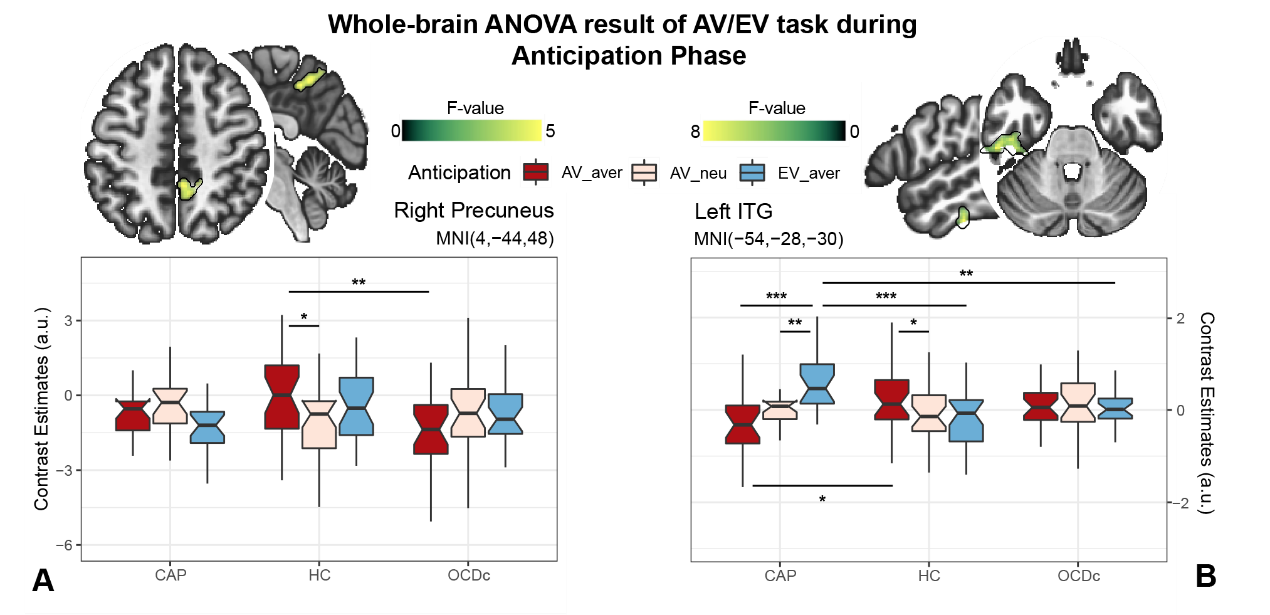


Supplementary Fig.4


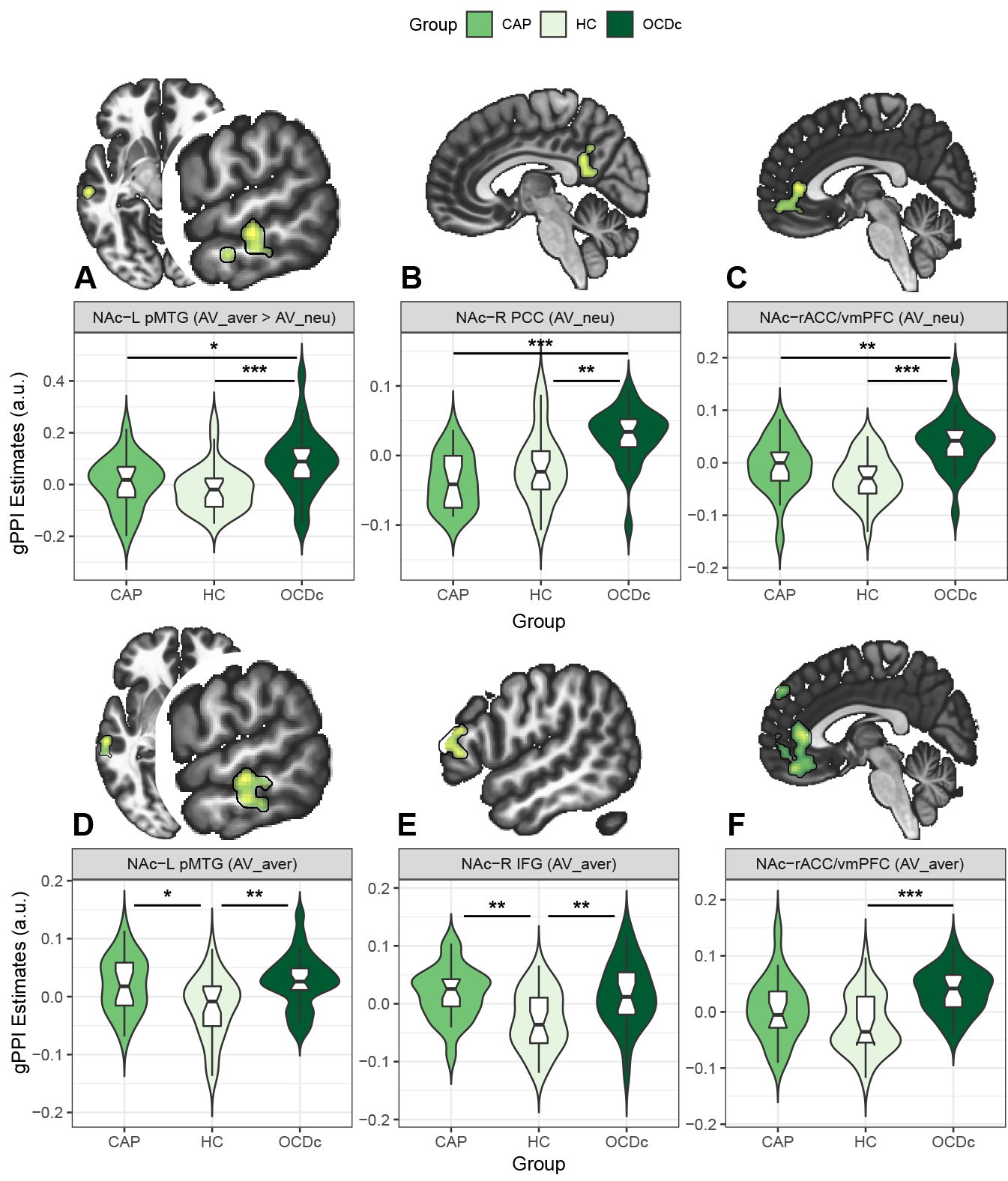


Supplementary Fig.5


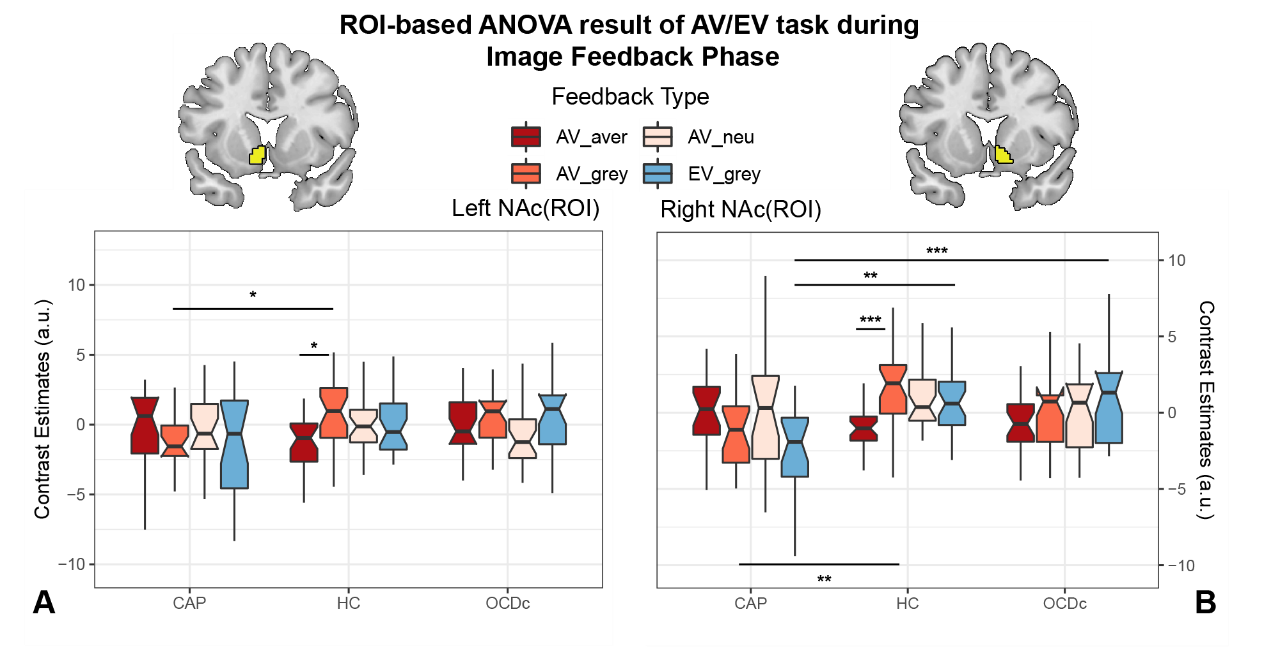


Supplementary Fig.6
